# Ivacaftor and sinonasal pathology in a cystic fibrosis patient with genotype delta F508/S1215N

**DOI:** 10.1186/2045-7022-5-S4-P2

**Published:** 2015-06-26

**Authors:** Maaike Berkhout, Charlotte Vreede, Arwen Sprij, Wytske Fokkens, Harry Heijerman

**Affiliations:** 1 Academic Medical Center, Department of Otorhinolaryngology, Amsterdam, Netherlands; 2 Haga Teaching Hospital, Department of Pulmonology, The Hague, Netherlands; 3 Haga Teaching Hospital, Department of Pediatrics, The Hague, Netherlands

## Background

Since the discovery of the cystic fibrosis (CF) gene in 1989, attempts have been made to develop a new therapeutic approach by targeting the underlying CFTR protein defect. Ivacaftor (Kalydeco®, Vertex Pharmaceuticals) is the first of a new class of drugs known as CFTR protein potentiators. This drug is functional in “CFTR gating” or type III mutations, in which a dysfunctional CFTR protein is present at the apical membrane. Ivacaftor facilitates improved chloride transport by increasing the opening time of the CFTR channel 1. Research has shown that treatment with ivacaftor can significantly improve lung function by about 10% [1]. Adverse events of ivacaftor include upper respiratory tract infections, nasal congestion and headaches [2].

## Case report

In our case report we illustrate a positive effect of ivacaftor on the sinonasal pathology in a 17 year old patient with CF. Her CF genotype showed a heterozygous deltaF508/S1251N mutation, in which the S1251N mutation is a type III mutation and can therefore be influenced by ivacaftor. In addition to her pulmonary symptoms she chronically suffered from headaches and nasal obstruction, most likely caused by chronic rhinosinusitis. During these complaints two CT-sinuses were performed (fig. 1A and 1B), showing opacification of all paranasal sinuses. Despite accurate treatment of the rhinosinusitis, with nasal irrigations, nasal steroids complaints persisted. After 5 months of ivacaftor use, a new CT-sinus (fig. 1C) showed complete resolution of the opacification of the paranasal sinuses and a decrease in symptoms of sinonasal disease. This positive effect of ivacaftor on sinonasal pathology seems promising, therefore more research is needed to evaluate the effect of ivacaftor on the upper airways in CF.

## Consent

Written informed consent was obtained from the patient for publication of this abstract and any accompanying images.
